# Supplementary material for: Content-rich biological network constructed by mining PubMed abstracts
Source: BMC Bioinformatics. 2004 Oct 8;5:147. doi: 10.1186/1471-2105-5-147 (PMC528731; doi:10.1186/1471-2105-5-147)
Supplement: Additional File 2 — The original results of the above study (non-essential files are deleted to keep the file size under the limit set by BMC bioinformatics). [file 1471-2105-5-147-S2.bz2 › chilibotAdditionalFile2/dip10/371ID10950949E6046/html/FGF2_FGFR1.html]

 


 **FGF2** and **FGFR1** 
  
Found 575 abstracts in PubMed, retrieved 10.  
 

 What does Google say? 
 PDF only 
| .edu only 

---

**Interactive relationship** (e.g. stimulation, inhibition, etc)

**Stimulatory relationship**- In situ hybridization analyses of TRalpha knockout mice, which have impaired ossification and skeletal mineralization, revealed reduced  **FGFR1**  mRNA expression in osteoblasts and osteocytes, whilst T3 failed to stimulate  **FGFR1**  mRNA or enhance  **FGF2**  activated MAPK signaling in TRalpha null osteoblasts.  Ref: 12805413 Mol Endocrinol< MedlineTA>Mol Endocrinol,
- Analysis of tryptic phosphopeptide maps of FGFR 1  [ **FGFR1** ]  from cells stimulated with FGF 2  [ **FGF2** ]  alone and FGF 2  [ **FGF2** ]  together with heparin showed that FGF 2  [ **FGF2** ]  alone stimulated a several fold increase in tyrosine 463 in the juxtamembrane domain.  Ref: 12799194 Exp Cell Res, 2003
**Neutral relationship**

**Non-interactive relationship** (e.g. studied together, co-existance, homology, etc.)

- METHODS The expression of VEGF, FLK 1, bFGF  [ **FGF2** ]  and FLG  [ **FGFR1** ]  in 40 young and 30 postmenopausal women with breast cancer was studied by immunohistochemical method SABC, with its relation with axillary lymph node metastasis and the clinical and pathologic characteristics.  Ref: 12795839 Zhonghua Zhong Liu Za Zhi, 2003
- We also measured components of the FGF 2  [ **FGF2** ]  ternary signaling complex, fibroblast growth factor receptor 1  [ **FGFR1** ]  FGFR 1  [ **FGFR1** ]  and syndecan 4.  Ref: 12832283 Am J Physiol Lung Cell Mol Physiol< MedlineTA>Am J Physiol Lung Cell Mol Physiol, 2003
- Similarly, T3 enhanced  **FGF2**  activated autophosphorylation of  **FGFR1** , but did not modify  **FGF2**  induced phosphorylation of the docking protein FRS2.  Ref: 12805413 Mol Endocrinol< MedlineTA>Mol Endocrinol, 2003
- There was also a significant difference in VEGF, bFGF  [ **FGF2** ] , FLK 1, FLG  [ **FGFR1** ]  and MVC between the stage 0 approximately II and stage III approximately IV P <.  Ref: 12795839 Zhonghua Zhong Liu Za Zhi, 2003
- We have studied the mechanism whereby heparin directs FGF 2  [ **FGF2** ]  induced FGF receptor 1 FGFR 1  [ **FGFR1** ]  signal transduction.  Ref: 12799194 Exp Cell Res, 2003
- FGF 2  [ **FGF2** ]  alone stimulated maximal phosphorylation of Src homology domain 2 tyrosine phosphatase SHP 2 and the adaptor molecule Crk, in heparan sulfate deficient Chinese hamster ovary CHO 677 cells expressing FGFR 1  [ **FGFR1** ] .  Ref: 12799194 Exp Cell Res, 2003
- The mean expression of VEGF, bFGF  [ **FGF2** ] , FLK 1 and FLG  [ **FGFR1** ]  of axillary lymph node positive patients was higher than the negative ones both in young and postmenopausal women groups P <.  Ref: 12795839 Zhonghua Zhong Liu Za Zhi, 2003
- Immunohistochemical assays for bFGF  [ **FGF2** ] , fibroblast growth factor receptor 1  [ **FGFR1** ]   **FGFR1** , and proliferating cell nuclear antigen were performed.  Ref: 12784342 Cancer, 2003
- These changes are associated with specific alterations in the regulation of FGF 2  [ **FGF2** ] , FGFR 1  [ **FGFR1** ]  and syndecan 4 in the airway epithelial mesenchymal trophic unit, which may be associated with the developmental problems of the lung associated with exposure to ozone.  Ref: 12832283 Am J Physiol Lung Cell Mol Physiol< MedlineTA>Am J Physiol Lung Cell Mol Physiol, 2003
